# Lightweight NiFe_2_O_4_ with controllable 3D network structure and enhanced microwave absorbing properties

**DOI:** 10.1038/srep37892

**Published:** 2016-11-29

**Authors:** Fen Wang, Xing Wang, Jianfeng Zhu, Haibo Yang, Xingang Kong, Xiao Liu

**Affiliations:** 1 School of Materials Science and Engineering, Shaanxi University of Science and Technology, Weiyang, Xi’an, Shaanxi 710021, PR China

## Abstract

3D network structure NiFe_2_O_4_ was successfully synthesized by a templated salt precipitation method using PMMA colloid crystal as templates. The morphology, phase composition and microwave absorbing properties of as-prepared samples were characterized by scanning electron microscopy (SEM), X-ray diffraction (XRD), vector network analyzer (VNA), and so on. The results revealed that the 3D network structure was configurated with smooth spherical walls composed of NiFe_2_O_4_ nanocrystals and their pore diameters being in the range of 80–250 nm. The microwave absorption properties of the 3D network structure NiFe_2_O_4_ were crucially determined by the special structure. The synergy of intrinsic magnetic loss of magnetic NiFe_2_O_4_ and the interfacial polarization enhanced by 3D network structure and the interaction of multiple mechanisms endowed the sample with the feature of strong absorption, broad bandwidth and lightweight. There is more than one valley in the reflection loss curves and the maximum reflection loss is 27.5 dB with a bandwidth of 4 GHz. Moreover, the 3D network structure NiFe_2_O_4_ show a greater reflection loss with the same thickness comparing to the ordinary NiFe_2_O_4_ nanoparticles, which could achieve the feature of lightweight of the microwave absorbing materials.

Very recently, electromagnetic interference (EMI) as a new way of pollution has attracted people’s great attention, which results from the explosive growth in application of electronic devices, radar systems and local area networks^1^,^2^. In order to protect the wireless devices and the biological systems from threaten of the EMI, materials with microwave absorbing properties were proposed.

Numerous microwave absorbing materials have been explored during the latest years and the microwave absorbing materials are now expected to possess the feature of strong absorption, broad band and lightweight. It is well known that the absorbing property is closely related to the electromagnetic parameter, impedance match and the microstructures^3^,^4^. Over the past few years, extensive studies on materials with the special microstructures have been done to enhance the microwave absorbing properties, including yolk-shell structures^5^, hollow structures^6^, porous structures^7^,^8^ and magnetic/carbon composites^9^. Among these candidates, porous and hollow structures were taken for the most ideal options due to their features of strong absorption, broad bandwidth and lightweight. Particularly, the three-dimensional ordered porous structure materials, often accompanied by a hierarchical structure, are now attracting much attention not only for its fascinating structure but also promising applications in various fields such as gas sensors^10^,^11^, catalyzer^12^,^13^, Energy transformation^14^,^15^ etc. Simultaneously, It can also be adopted as thermal insulation layer in virtue of its low thermal conductivity^16^. Zhao *et al.* proposed a honeycomb SnO_2_ microwave absorbing material with an excellent dielectric loss and the optimal reflection loss (RL) is −37.6 dB obtained at 17.1 GHz with thin thickness of 2.0 mm^17^. As far as we know, the soft magnetic nickel ferrite with a 3D network structure used as microwave absorber has never been reported. The soft magnet nickel ferrite have outstanding performance due to its large saturation magnetization, obvious Snoek’s limit, less coercivity, excellent chemical stability and corrosion resistance, which result in its immense potential application in microwave absorbing field^18^,^19^. Nevertheless, the high density and limited absorption bandwidth could not meet the requirements of facilities for light electromagnetic wave absorbing materials. Gu *et al.* introduced a mesoporous NiFe_2_O_4_ material that had high surface areas, tunable pore sizes, and periodic pore arrangements, showing optimal reflection loss of −22.5 dB with a band width less than 2 GHz^20^.

In this work, a templated salt precipitation method was adopted to prepare the 3D network structure nickel ferrite. The obtained 3D network structure NiFe_2_O_4_ materials with the configuration of ordered meshes exhibit an excellent microwave absorption performances due to the match of magnetic loss and dielectric loss.

## Results and Discussion

It has been studied that metal precursor involving polar solvents can infiltrate the colloidal crystals templates more completely, and introducing fewer structural defects during the composite formation^21^. Therefore, the infiltrating degree of the template and solvent is vital for the microstructure. Figure S2 shows the contact angle of the PMMA template with ethylene glycol and the result indicates that PMMA has superior wettability with the solvent. Simultaneously, PMMA microspheres dissolve slightly from the free surface in ethylene glycol, releasing void space for the crystal growth. In the early stage of the work, we prepared some PS templated nickel ferrite which possesses discrete and disordered pore structure as shown in Fig. S3. We also explored the microwave absorbing performance of the PS templated nickel ferrite which showed similar performance with that of the NiFe_2_O_4_ NPs. Therefore, we didn’t discuss this part anymore.

**Figure 1:**
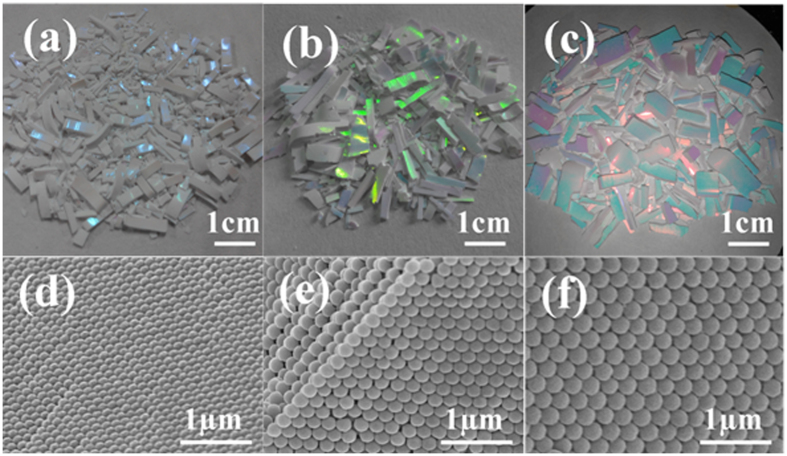
Photo (**a**–**c**) and FE-SEM (**d–f**) of the PMMA templates with different microsphere diameter (**a**,**d**) 160 nm, (**b**,**e**) 220 nm and (**c**,**f**) 280 nm, respectively.

Templates based on neat array are the basis of the integrated 3D network structure. Figure 1 shows the microstructures and photographs of the PMMA colloidal crystals templates (CCTs). As shown, the uniformly-sized PMMA microspheres are arranged in a face-centered cubic structure (Fig. 1d–f). The flamboyant iridescence reflected on the surface of CCTs originates from the alternately arranged microspheres and air (Fig. 1a–c). In addition, the iridescence color wavelength shows red shift as the microsphere diameter increases. The relationship between maximum iridescence color wavelength and microsphere diameter is discussed in detail in Fig. S4.

**Figure 2:**
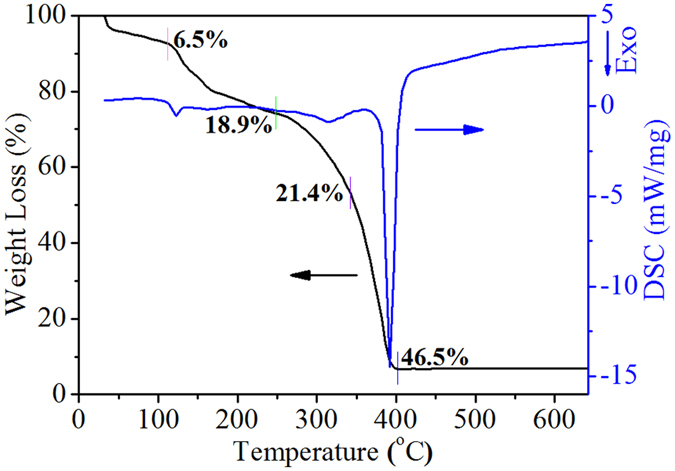
TG-DSC curves of PMMA CCTs filled with EG solution of Fe(NO_3_)_3_ and Ni(NO_3_)_3_ in simulation air with N_2_/O_2_ volume ratio of 8:2.

In order to investigate the thermal behavior of PMMA CCTs filled with EG solution, the calcination process was analyzed by TG-DSC curves in Fig. 2. The first 6.5% weight loss around 100 °C and the second 18.9% weight loss before 240 °C occur mainly corresponding to the solvent evaporation and the release of NO_x_ gas. Simultaneously, the correlative exothermic peak on the DSC curve is related to the formation of glyoxylate^22^. The third stage with an exothermic peak on the DSC curve at 320 °C is ascribed to the oxidation-decomposition of glyoxylate. And the last 46.5% weight loss with a sharp exothermic peak at 350–400 °C is associated with the oxidation-decomposition of the PMMA templates^23^. Meanwhile, the weight decrease continues to ca. 400 °C with the total weight loss of 93.3%, indicating the formation of NiFe_2_O_4_. According to TG-DSC measurements, it could be determined that the minimum feasible template-removing temperature is 400 °C.

The obvious characteristic peaks are perfectly indexed to the nickel ferrite spinel phase (JCPDS card No. 54–0964), which confirms the formation of NiFe_2_O_4_ at least 500 °C (Fig. S5). Additionally, there is no disturbance in the patterns, which means high purity of the products. Moreover, the peaks become sharper with the increasing of temperature, which indicates the growing of NiFe_2_O_4_ NPs. Subsequently in Fig. S6, the average crystallite size D are estimated from peak widths using the Debye-Scherrer’s formula: 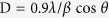
24. It is significant to find the appropriate template-removing temperature because the temperature also affects the formation of NiFe_2_O_4_.

**Figure 3:**
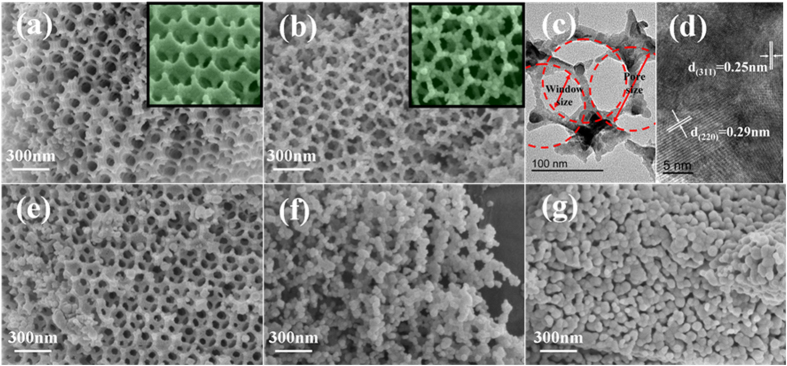
FE-SEM (**a**,**b**,**e**–**g**) and HR-TEM (**c**,**d**) of the samples calcined at different temperatures (**a**) 500 °C, (**b**–**d**) 600 °C, (**e**) 700 °C, (**f**) 800 °C, (**g**) 900 °C, respectively. (The microsphere diameter of the templates is 200 nm).

Figure 3 shows the microstructures of obtained samples calcined at different temperatures. The well-calibrated circular meshes, originated from the templates removal, keep a relative long-range ordered arrangement, which is similar to the face-centered cubic (FCC) structure of the PMMA CCTs. Among the periodic meshes, there are windows interconnected in three dimensions involving many layers. Moreover, the inset magnification areas show smooth, spherical appearance of the walls in obtained samples and usually more precursor solution exist in the gaps of the microspheres that promontories occur after the calcination (Fig. 3a,b). TEM images show a close-up look of the wall and the pore size and window size are measured to 150 and 57 nm by using PMMA CCT with the diameter of 200 nm. The d-spacing of 0.25 and 0.29 nm are corresponding to the NiFe_2_O_4_ (311) and (220) lattice planes, respectively (Fig. 3c,d). From the above, it can be concluded that the 3D network structure NiFe_2_O_4_ has been successfully synthesized. However, the wall thickness is closely related to the particle size and the wall thickness turns thicker with NiFe_2_O_4_ the increasing of the temperature (Fig. 3a,b,e–g). The textured wall skeletons are typically constructed by NiFe_2_O_4_ NPs with the diameters of 10–50 nm which is obtained by the randomly statistical calculation (50 nanocrystals).With the increasing of the calcination temperature, some nanocrystals aggregate destroying the orderliness in regions and when the crystallite size exceeds ca. 40 nm, the 3D network structure breaks down (Fig. 3f,g) This result is identical with the previous reports^21^,^22^.

**Figure 4:**
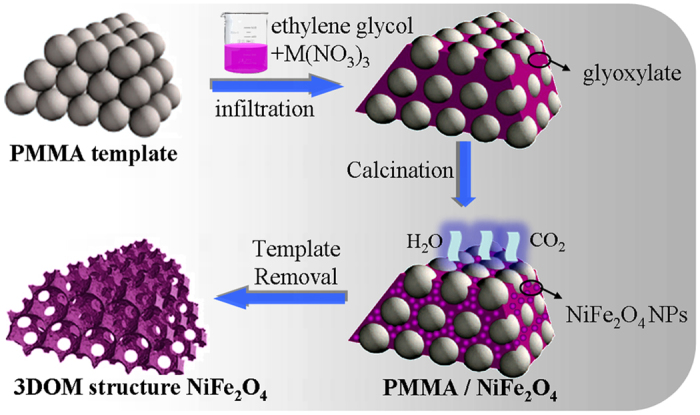
Formation mechanism of the 3D network structure NiFe_2_O_4_ samples.

Based on the above results, we propose a formation mechanism of the 3D network structure NiFe_2_O_4_ in Fig. 4. The large scale ordered arrangement of the CCTs determines the 3D network structure of NiFe_2_O_4_ samples. Both the PMMA microspheres and meshes of 3D network show a FCC structure in three-dimensional space (Figs 1 and S6). The NiFe_2_O_4_ NPs result from the oxidation-decomposition of glyoxylate and the meshes take shape after template removal (Fig. 2). In this process, softening PMMA microspheres are squeezed by growing grains which results in the smaller pore size compared with the sphere diameter. Overall, the pore size is closely related to both the NiFe_2_O_4_ grain size and PMMA sphere diameter. Moreover, the calcination temperature plays an important role because the 3D network structure NiFe_2_O_4_ is constructed by NiFe_2_O_4_ NPs and the NiFe_2_O_4_ nanocrystalline grains grow up as the increasing of calcination temperature (Figs S5 and S6). However, there is a limiting NP size for 3D network structure to avoid collapse of the structure (Fig. 3). The results indicate that the selected calcination conditions (2 h at 500 °C, 600 °C and 700 °C) are feasible and the excessively high temperature should be avoided in the template removal process.

To further investigate the microwave absorbing properties of the 3D network structure NiFe_2_O_4_, the reflection loss (RL) is calculated by using equations (1)^9^,^25^.


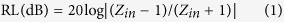


In which Z_*in*_ is the input impedance of the absorber and the normalized input Z_*in*_ was calculated by equations (2).


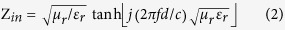


**Figure 5:**
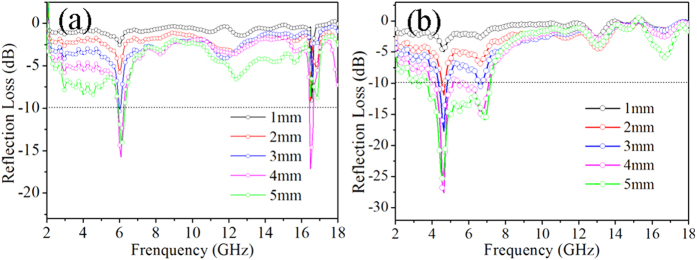
Frequency dependence of reflection loss of the ordinary NiFe_2_O_4_ NPs (**a**) and the 3D network structure NiFe_2_O_4_ samples (**b**) by varying the thickness of the absorbent.

Where *ε*_*r*_ and *μ*_*r*_ is the complex relative permittivity and permeability, respectively, *f* is the frequency, *d* is the thickness of the absorption layer and *c* is the propagation velocity of the electromagnetic wave in vacuum. Synchronously, Fig. 5 shows the frequency dependent of calculated reflection loss (RL) curves at different specimen thicknesses. It is observed that there are three valleys with the bandwidth at 4.0–5.7 GHz, 6.2–8.2 GHz and 12.0–14.0 GHz and the maximum reflection loss is 27.5 dB with a bandwidth of 4 GHz for the 3D network structure NiFe_2_O_4_ with a thickness of 4.0 mm, which is stronger and wider than the ordinary NiFe_2_O_4_ NPs. For better comparison, the minimum reflection loss and the absorption bandwidth of some microwave absorbing materials are listed in Table 1. To our knowledge, the minimum reflection loss of the 3D network structure NiFe_2_O_4_ (RL = −27.5 dB) and the absorption bandwidth (4 dB) for this work are better than those of the similar microwave absorbing materials which implies that the as-prepared 3D network structure NiFe_2_O_4_ can be potentially used in the microwave absorbing field.

Moreover, the 3D network structure NiFe_2_O_4_ show a greater reflection loss with the same thickness comparing to the ordinary NiFe_2_O_4_ nanoparticles. In other words, to meet the same performance, less 3D network structure NiFe_2_O_4_ will be required in the applications, which demonstrates its feature of lightweight. Most often, reflection loss less than 10, 20 decibel (dB) is equivalent to 90% and 99% attenuation, respectively. The multiple valleys can be attributed to the special 3D network structure and the reasons are introduced in following discussions.

**Figure 6:**
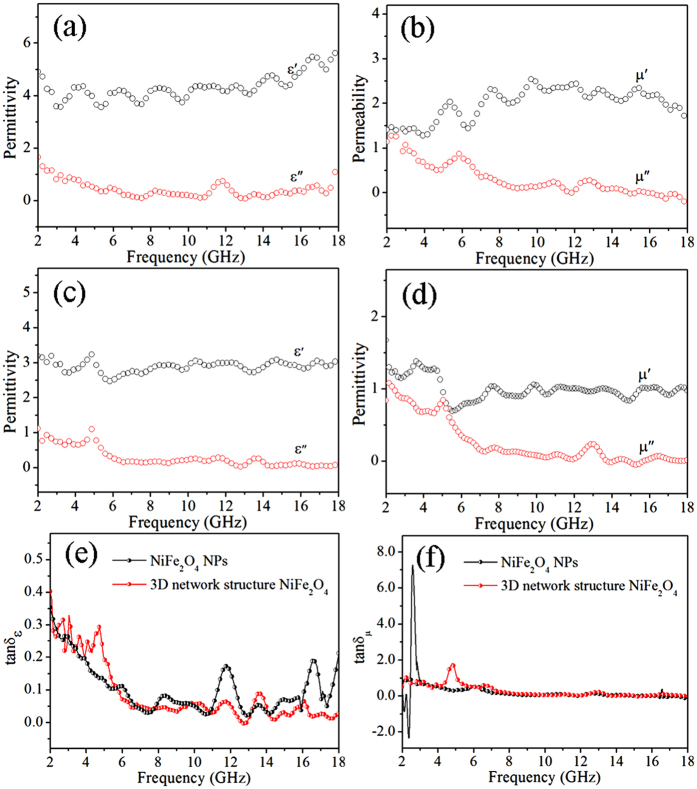
Variation of complex permittivity and complex permeability for the ordinary NiFe_2_O_4_ NPs (**a**,**b**) and the 3D network structure NiFe_2_O_4_ samples (**c**,**d**) and loss tangent (**e**,**f**) in 2–18 frequency regions.

It is well known that the microwave absorption property is mainly determined by the electromagnetic parameter, the impedance match and the microstructure. As a consequence, the frequency dependence complex permittivity (*ε*_*r*_) and complex permeability (*μ*_*r*_) of ordinary NiFe_2_O_4_ NPs (Fig. 6a,b) and 3D network structure NiFe_2_O_4_ samples (Fig. 6c,d) were recorded to investigate the electromagnetic properties of NiFe_2_O_4_/paraffin composites by using a coaxial reflection/transmission method. Generally, the real parts (*ε*′ and *μ*′) represent the storage capability of electric and magnetic energy, whereas the imaginary parts (*ε″* and *μ″*) stand for the inner dissipation abilities, respectively^26^. As shown in Fig. 6c, the real part and the imaginary part of complex permittivity of the 3D network structure follow the similar trend with the increasing frequency. Moreover, an obvious peak occurs at 5 GHz and the fluctuation indicating a resonance behavior. Whereas the imaginary part of complex permeability (*μ*″) exhibits one major peak at around 5 GHz and a minor peak at *f* = 13 GHz, however the corresponding real part (*μ*′) decreases in the same region. By contrast, both *ε*′ and *μ*′ of the NiFe_2_O_4_ NPs is larger than the 3D network structure NiFe_2_O_4_ on value, but *ε*″ and *μ*″ of the NiFe_2_O_4_ NPs and the 3D network structure NiFe_2_O_4_ are equal. Figure 6e,f show the comparison of the dielectric loss tangent and magnetic loss tangent, respectively. Both the ordinary NiFe_2_O_4_ NPs and the 3D network structure NiFe_2_O_4_ samples are equipped with the weak magnetic loss.

The magnetic properties of the 3D network structure NiFe_2_O_4_ powders were also investigated with hysteresis loops (Fig. S8). The saturation magnetizations, coercivities, and remnant magnetizations increase gradually with the increase of the annealing temperature but the coercivities are still too small that can be ignored. Generally, the reasons for magnetic loss include hysteresis, domain wall resonance, eddy current effect and the natural ferromagnetic resonance^27^. The contribution of domain wall resonance is negligible since it occurs usually in the low frequency about 1–100 MHz range which is far lower than the measurement range. The eddy current loss is related to the diameter of the nanoparticles (*d*) and electric conductivity (*σ*), which can be expressed by the following equation^28^:


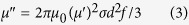


In Eq. (3), *μ*_*0*_ is the permeability of vacuum. As a deformation formula:


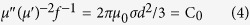


According to the skin-effect criterion, if the magnetic loss only originates from the eddy current loss, the values of *μ*″(*μ*′)^−2^*f*^−1^ should be constant when the frequency is changed. The C_0_ value is shown in Fig. S9 and it changes drastically as a function of frequency at the frequency range of 2.0–8.0 GHz. Therefore, it can be concluded that the magnetic loss at 5 GHz is caused by the natural-resonance. However, when *f* > 13.0 GHz, the section is approximately constant, which is ascribed to eddy current effect. That may issue from the agglomeration of small NiFe_2_O_4_ nanocrystals.

In addition to the magnetic loss, the dielectric loss is crucially determined by the configuration of the 3D network structure. It is easy to understand that dipolar polarization takes place in 3D network structure NiFe_2_O_4_ due to the small NiFe_2_O_4_ nanoparticles, which can be considered as dipoles. Additionally, numerous voids existing in the 3D network structure NiFe_2_O_4_ samples cause multiple reflections and scatter in the material, at the meantime, provide more interfaces between paraffin, NiFe_2_O_4_ nanocrystallines and air, which induce interactions of dipoles and interfacial polarization which contribute to the dielectric loss significantly^29^.

**Figure 7:**
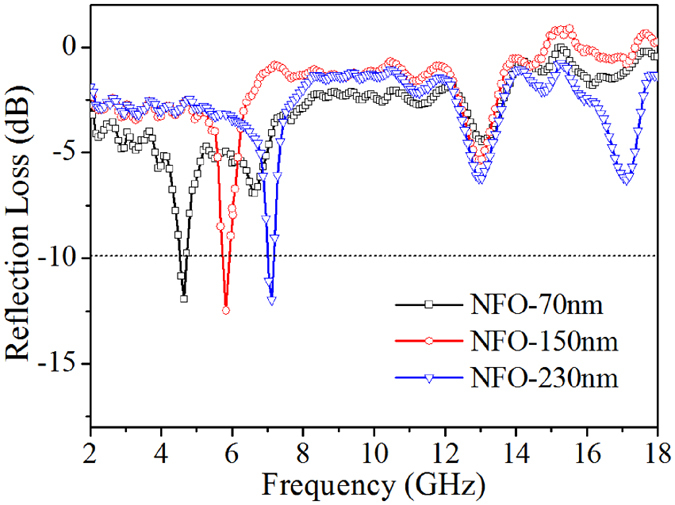
Frequency dependence of reflection loss of the 3D network structure NiFe_2_O_4_ samples with different pore size. (NFO represents NiFe_2_O_4_ and the thickness is all of 3 mm).

Multiple mechanisms work together changing the position of reflection loss peak as shown in Fig. 7. Based on the fact that the skeletons of 3D network structure NiFe_2_O_4_ are constituted by nanocrystalline domains possessing different orientations, it could be proposed that the energy levels of 3D network structure NiFe_2_O_4_ are discontinuous according to quantum effects^30^. When the energy originating from microwave radiation can match the energy level, an electron can absorb a wavicle, hopping from a lower energy level to a higher one. Energy of electromagnetic wave will be attenuated gradually. These results indicate that the absorption peak positions and frequency ranges can be regulated easily by adjusting the pore diameters of the network structures, and thus a broadband absorption can be designed using a multilayered absorbing structure.

The reasons for the excellent microwave absorption properties of 3D network structure NiFe_2_O_4_ are concluded as follows: firstly, magnetic loss was attributed to the natural resonance mechanism (intrinsic magnetic loss) of NiFe_2_O_4_. Secondly, dipole polarizations and interfacial polarization were presented due to the interfaces between NiFe_2_O_4_ nanoparticles, air and paraffin wax. The complementarities between multiple mechanisms also played an important role in improving the microwave absorption properties. In consequence, 3D network structure NiFe_2_O_4_ studied here are promising candidates for application as microwave absorber and these 3D network structure can also be used not limited to ferrites.

## Conclusions

3D network structure NiFe_2_O_4_ was successfully synthesized by using PMMA colloidal crystal templates for its better wettability with polar solvents. From the thermal behavior of PMMA/NiFe_2_O_4_ composites, the optimum temperature to remove CCTs was determined no less than 400 °C. Anyhow, excessively high temperature must be avoided because nanocrystalline with size more than 40 nm will make the 3D network structure collapse. As-prepared NiFe_2_O_4_ samples presented a perfect 3D network structure that built by nanocrystalline and the interconnection meshes involved many layers. Intrinsic magnetic loss of NiFe_2_O_4_, interfacial polarization and the multi-reflection caused by 3D network structure made the sample show an excellent microwave absorbing properties and the reflection loss curves had three valleys. The minimum reflection loss is −27.5 dB with a thickness of 4.0 mm. Therefore, the prepared 3D network structure NiFe_2_O_4_ has potential applications in microwave absorbing field.

## Methods

The reagents mentioned were all of analytical grade and used without further purification except methyl methacrylate (MMA). The MMA monomer was previously distilled under the reduced pressure to remove the inhibitor. All the water of the experiments mentioned before was deionized.

### Fabrication of ordered PMMA CCTs

Non-cross-linked, monodisperse PMMA spheres with various diameters ranged from 100 to 350 nm were synthesized using emulsifier-free emulsion polymerization according to literatures^23^. Firstly, 10 g of MMA monomer was blended with 120 ml of the deionized water in a 250 ml of three-necked flask by stirring, and then the mixture was heated to 75 °C in oil bath under a N_2_ gas atmosphere protection. After 30 min, 0.1 g KPS of initiator was added. Five hours later, monodisperse PMMA spheres were obtained. The resultant PMMA microspheres were rinsed for several times with the deionized water and close-packed into colloidal crystals by the vertical deposition before use.

### Preparation of 3D network structure NiFe_2_O_4_ powders

The preparation strategy of 3D network NiFe_2_O_4_ could be described as a templated salt precipitation method. In detail, Ni(NO_3_)_2_·6H_2_O and Fe(NO_3_)_3_·9H_2_O were dissolved in ethylene glycol with a cation ratio of 1:2 by continuously stirring at least 8 h, given the viscidity of ethylene glycol. And the total ion concentration was 1.8 M. Then, the pre-prepared PMMA CCTs were soaked in precursor solution completely for 2 h with the subsequent vacuum filtration operation. After an overnight drying step, NiFe_2_O_4_ with the 3D network structure was prepared by calcining at a template-removing appropriate temperature for 2 h in the air. And a slow heating rate (e.g., 2 °C/min) was imperative to keep the structure as ordered as possible, especially at the stages when the polymer template gasifies or combusts.

### Characterization

The morphologies, geometric parameters and lattice fringes were characterized by field emission scanning electron microscope (FE-SEM, Hitachi S4800) and transmission electron microscope (TEM, FEI Tecnai G2 F20 S-TWIN). The thermogravimetry (TG) and differential scanning calorimetry (DSC) were recorded on a thermal analyzer (STA449F3, Netzsch, Germany) with a heating rate of 10 °C/min in a flowing mixed gas of N_2_ and O_2_. The phase composition and purity were analyzed by X-ray diffractometer (XRD, Rigaku D/max 2200 pc) using Cu Kα radiation of wavelength λ = 0.15418 nm. Besides, magnetization measurements were performed on a vibrating sample magnetometer (VSM, Lakeshore 7304) and the magnetic hysteresis loop measurements were performed at 8 kOe at 300 K. The microwave absorbing properties of the material was estimated based on the coaxial transmission/reflection method. The electromagnetic parameters of the 3D network structure NiFe_2_O_4_ samples were recorded by vector network analyzer (VNA) with the samples uniformly mixed with paraffin wax in a mass ratio of 7:3, forming ferrite/paraffin composites (here, paraffin wax contained in the ferrite/paraffin composites was only used as binder) and then pressed into a toroidal shape at a preesure of 0.6 MPa (Φ_out_ = 7.00 mm, Φ_in_ = 3.04 mm). The total mass of sample used in test is a fixed value of 0.13 g.

## Additional Information

**How to cite this article**: Wang, F. *et al.* Lightweight NiFe_2_O_4_ with controllable 3D network structure and enhanced microwave absorbing properties. *Sci. Rep.* **6**, 37892; doi: 10.1038/srep37892 (2016).

**Publisher's note:** Springer Nature remains neutral with regard to jurisdictional claims in published maps and institutional affiliations.

## Supplementary Material

Supplementary Information

## Figures and Tables

**Table 1:** Minimum reflection loss and the absorption bandwidth of some microwave absorbing materials.

Materials	[RL(dB)]min	Absorption bandwidth (GHz) (dB ≤ −10)	Ref.
Nanosized CoFe_2_O_4_	−8.0	0	[31]
Cobalt-Zinc ferrites	−23.6	4.0	[32]
Magnetic porous Fe_3_O_4_/BaCO_3_	−23.8	4.0	[33]
Mesoporous crystalline NiFe_2_O_4_	−22.5	1.8	[20]
3D network structure NiFe_2_O_4_	−27.5	4.0	This work
