# Supplementary material for: Lightweight NiFe2O4 with controllable 3D network structure and enhanced microwave absorbing properties
Source: Sci Rep. 2016 Nov 29;6:37892. doi: 10.1038/srep37892 (PMC5126577; doi:10.1038/srep37892)
Supplement: Supplementary Information [file srep37892-s1.doc]

**Supporting Information**

Lightweight NiFe_2_O_4_ with controllable 3D network structure and enhanced microwave absorbing properties

Fen Wang***^a^*[[1]](#footnote-2)***, Xing Wang***^a^***, Jianfeng Zhu***^a^***, Haibo Yang***^a^***, Xingang Kong***^a^*** and Xiao Liu***^a^***

*^a^* School of Materials Science and Engineering, Shaanxi University of Science and Technology, Weiyang, Xi’an, Shaanxi 710021, PR China

*Corresponding Author:* Professor Fen Wang

E-mail address: [wangf@sust.edu.cn](mailto:wangf@sust.edu.cn).

Tel: +86-29-86168688

Fax: +86-29-86168688

**Preparation of the NiFe_2_O_4_ nanoparticles**

Firstly, a certain amounts of Ni(NO_3_)_2_·6H_2_O and Fe(NO_3_)_3_·9H_2_O were dissolved in 50 mL ethylene glycol keeping a same concentration with the precursor solution of the 3D network structure NiFe_2_O_4_. Then, the mixture was dried at a constant temperature of 180 ^o^C. Finally, the obtained powder was calcined for 2 h. Fig. S1 shows the SEM images of the scattered NiFe_2_O_4_ nanoparticles with the size around 30 nm.


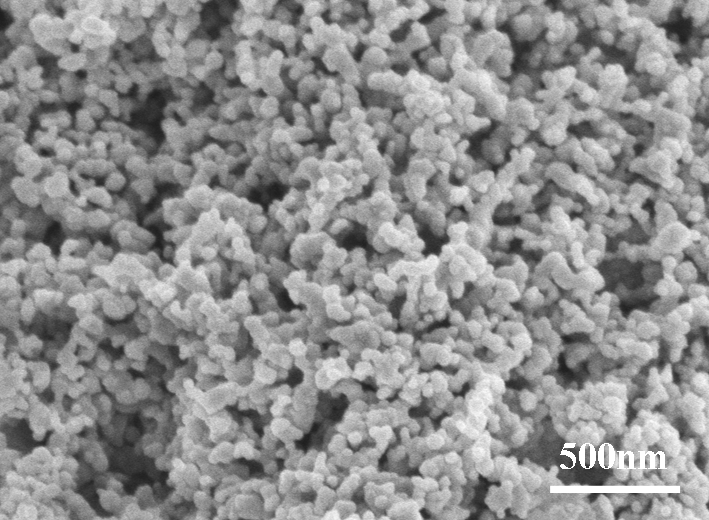


Fig. S1 SEM images of the NiFe_2_O_4_ nanoparticles

PMMA and PS (polystyrene) CCTs are both common organic templates. Figure S1 shows the contact angles of deionized water and ethylene glycol (EG) with films prepared by depositing PMMA/PS microspheres on glass sheets with thickness of ca. 20 µm. Synchronously, contact angles are automatically calculated from the Young-Laplace equation by fitting the liquid dropped shape [^1^](#_ENREF_1). Compared with the PMMA, both the water and EG have bigger contact angles on PS films. It indicates that PMMA has better wettability with polar solvents than PS which is conducive to the soaking process. Simultaneously, PMMA microspheres dissolve slightly from the free surface in ethylene glycol, releasing void space for the crystal growth. As a consequence, we employed PMMA as templates to ensure long-range ordered arrays of air spheres. PS templated nickel ferrite that possesses discrete and disordered pore structure was also presented in Fig. S3.


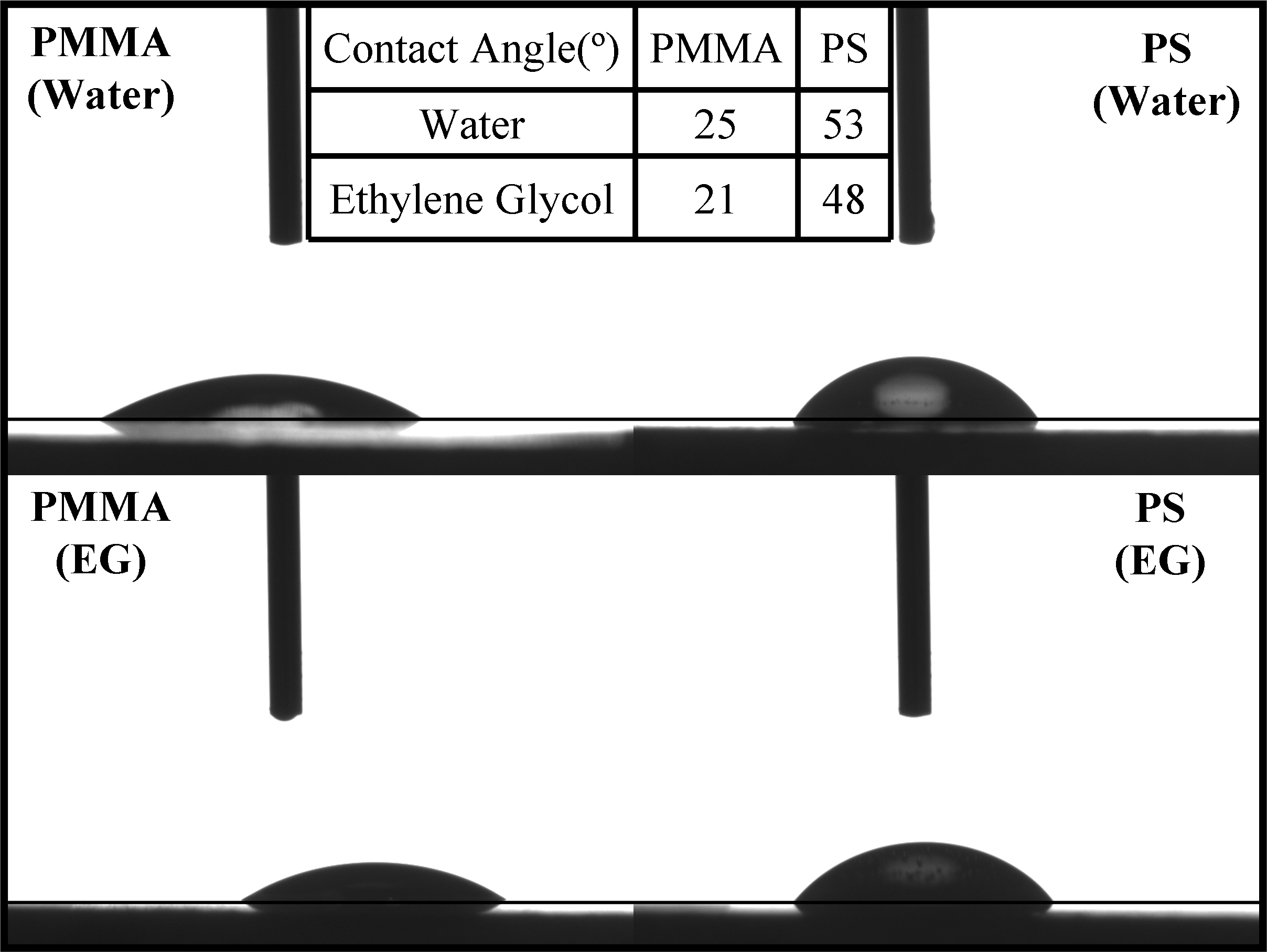


Fig. S2 Liquid contact angle pictures of PMMA and PS colloidal crystal templates.


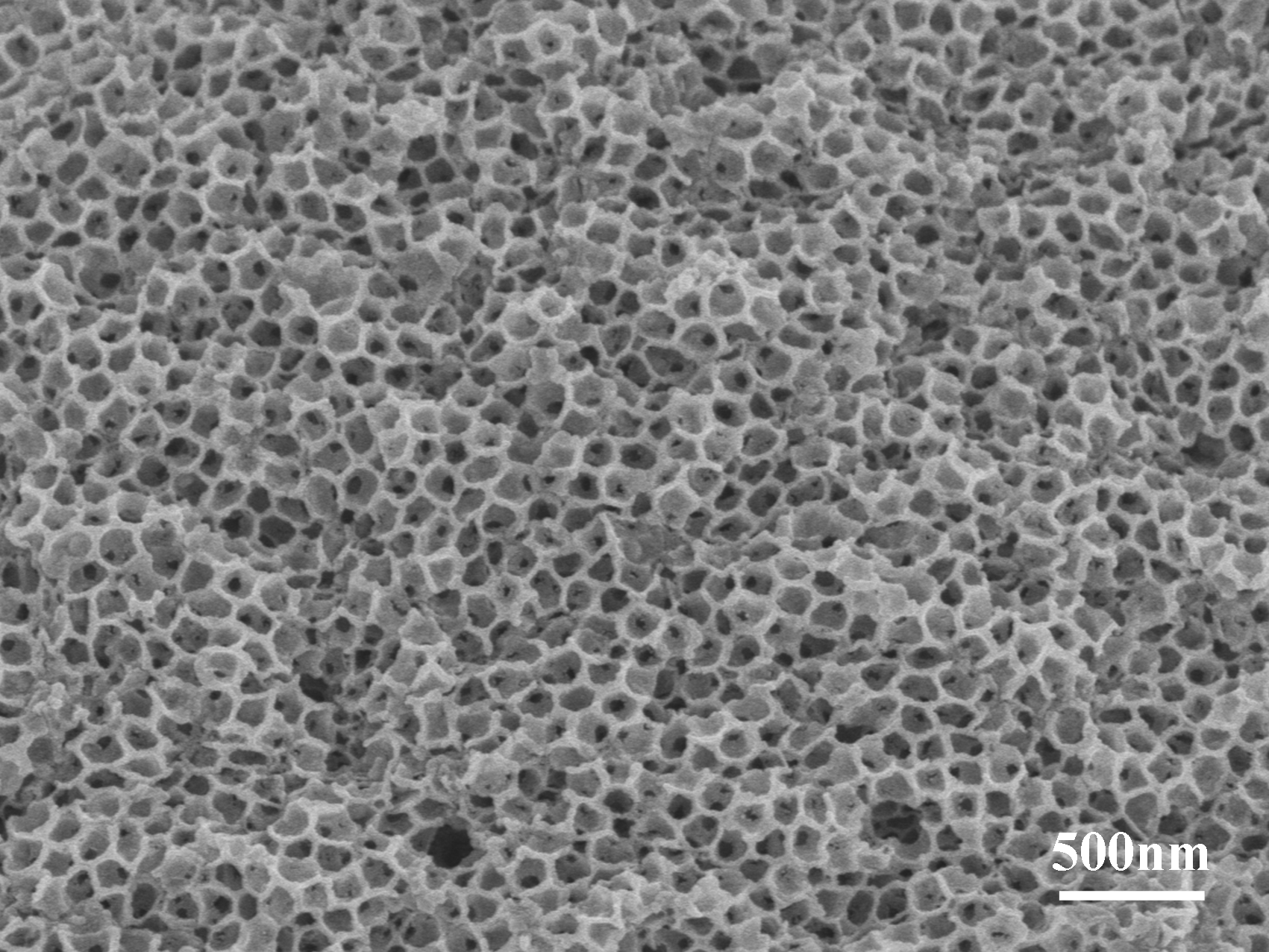


Fig. S3 SEM images of the PS templated nickel ferrite

The relationship between the stop band (maximum iridescence color wavelength) and the microsphere diameter can be explained by using the following equation [^2^](#_ENREF_2):

$\lambda=2\times\sqrt{2/3}\text{D}\times\sqrt{n_{1}^{2}f+n_{2}^{2}\left(1-f\right)}$ (1)

In Eq(1), $n_{1}$, $n_{2}$ are refractive indexes of PMMA and air, respectively. $f$ is the space occupancy of PMMA colloidal crystal template, ca. 0.74. $\lambda$, $\text{D}$ are the color wavelength and the microsphere diameter, respectively. The 3D ordered structure of PMMA CCTs can be further confirmed through the reflection spectra. As shown in Fig. S3, the calculated peak positions of the stop bands (maximum iridescence color wavelength) 446, 547 and 658 nm for the CCTs are assembled by PMMA microspheres in diameters of 180, 240 and 280 nm, respectively.


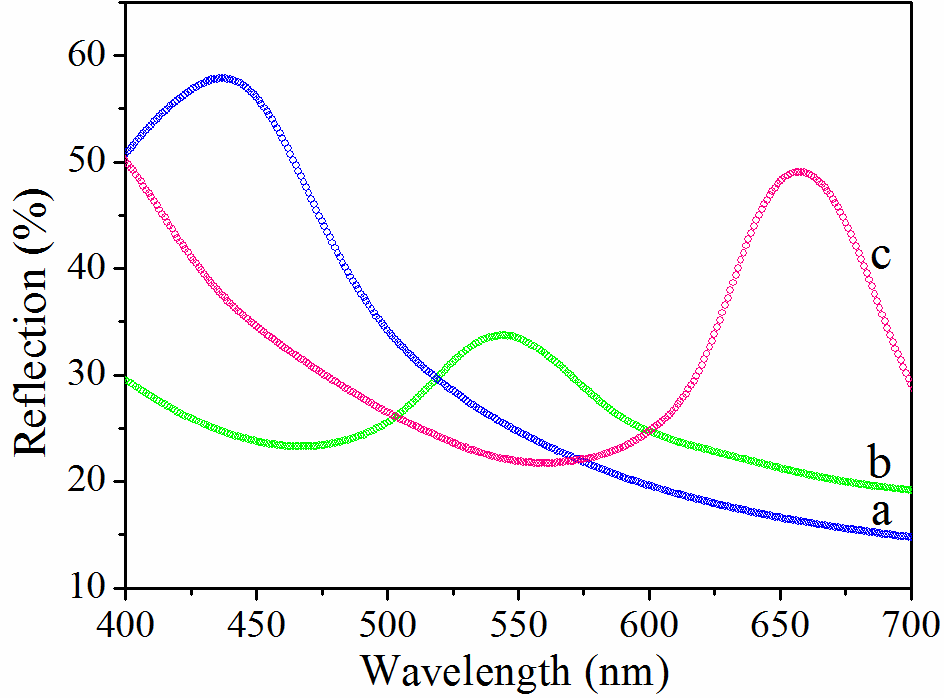


Fig. S4 Reflection spectra of PMMA films with the thickness of ca. 20 µm on glass sheets.


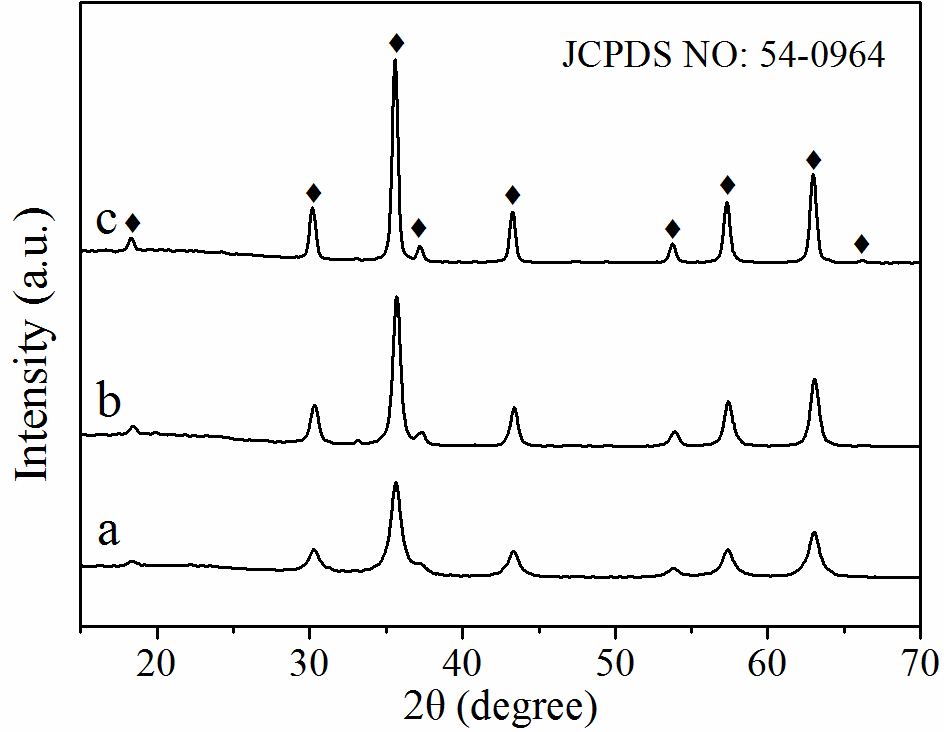


Fig. S5 XRD patterns of the samples calcined at different temperatures (a)500 ^o^C, (b)600 ^o^C, (c)700 ^o^C.


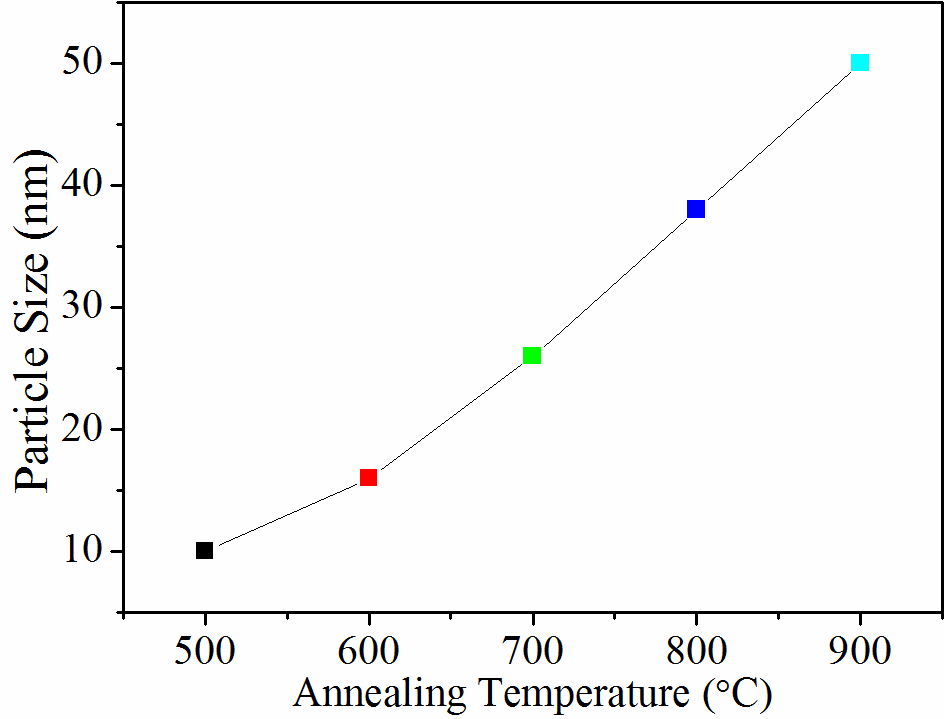


Fig. S6 The annealing temperature dependence particle sizes.


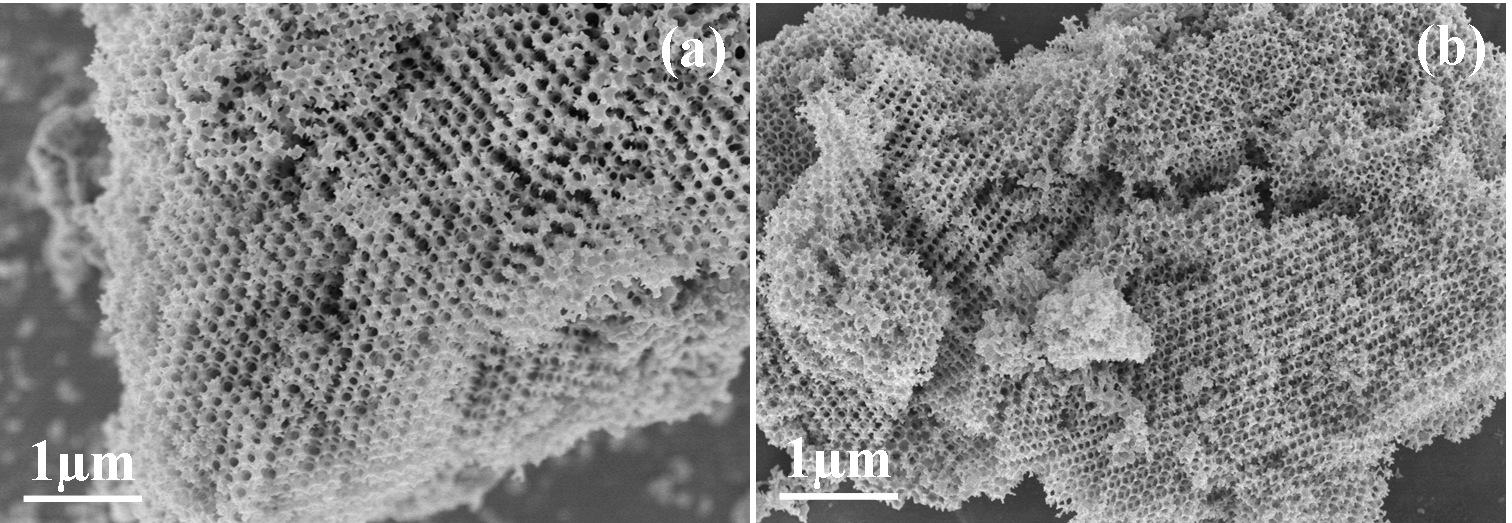


Fig. S7 SEM images of extensive scale 3D network structure with different pore sizes.


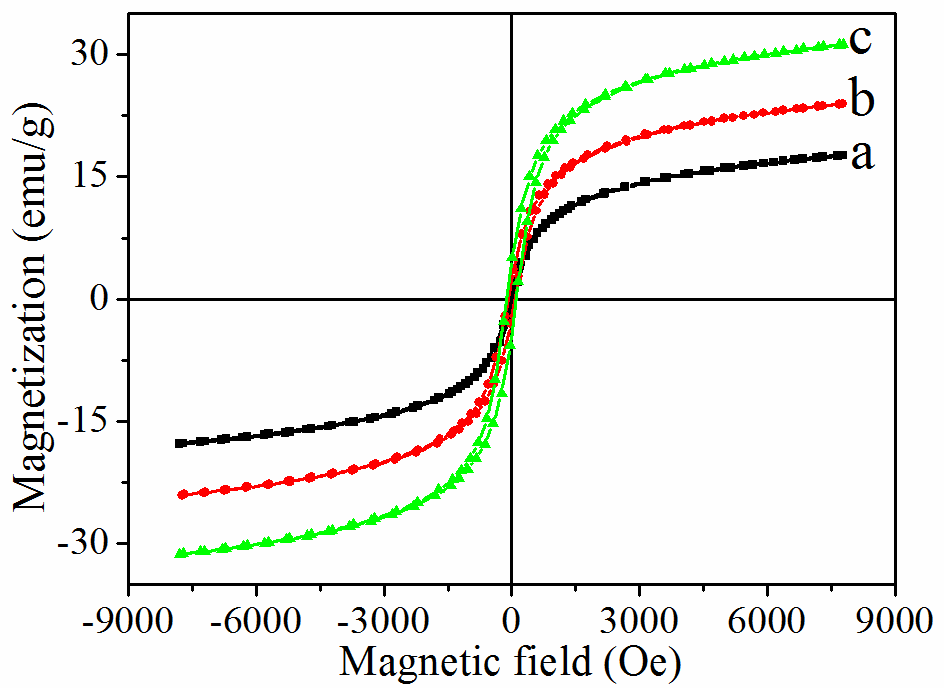


Fig. S8 Hysteresis loops of magnetic 3D network structure NiFe_2_O_4_ samples measured at 300 K, (a)500 ^o^C, (b)600 ^o^C, (c)700 ^o^C


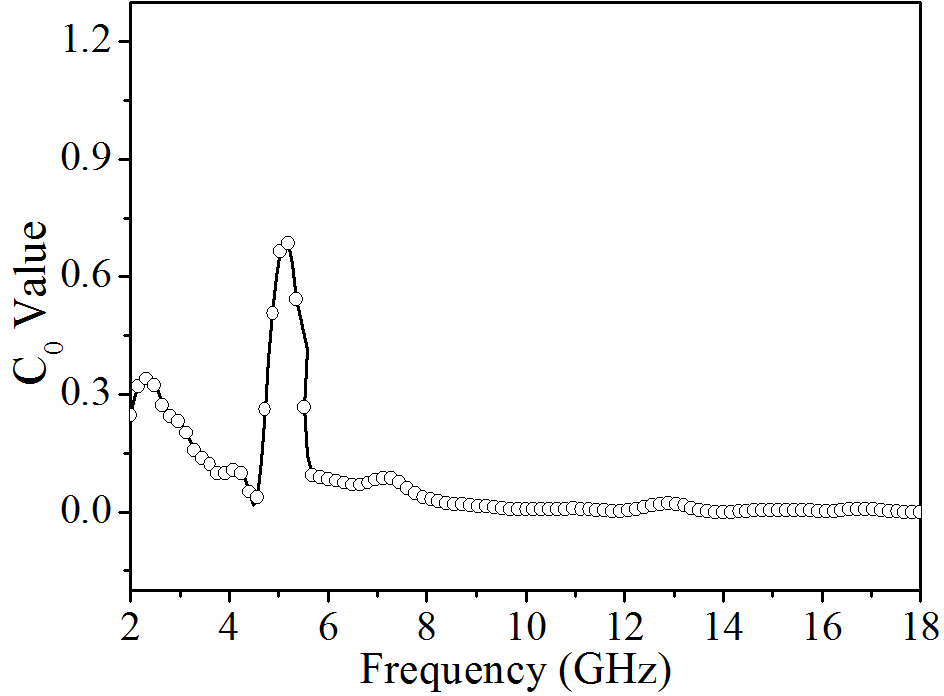


Fig. S9 The value C_0_ () of 3D network structure NiFe_2_O_4_ sample as a function of frequency.

**Reference**

1 Khayet, M., Álvarez, M. V., Khulbe, K. & Matsuura, T. Preferential surface segregation of homopolymer and copolymer blend films. *Surf. Sci.* **601**, 885-895 (2007).

2 Tang, B., Wu, C., Lin, T. & Zhang, S. Heat-resistant PMMA photonic crystal films with bright structural color. *Dyes. Pigments.* **99**, 1022-1028 (2013).

1. ^^ Corresponding author. Tel: +86-29-86168688; Fax: +86-29-86168688; Email: [wangf@sust.edu.cn](mailto:wangf@sust.edu.cn). [↑](#footnote-ref-2)
